# The genome sequence of the Small Dotted Buff moth, *Photedes minima *Haworth, 1809

**DOI:** 10.12688/wellcomeopenres.24264.1

**Published:** 2025-06-02

**Authors:** Douglas Boyes, Liam M. Crowley, Timilehin Adewumi, University of Oxford and Wytham Woods Genome Acquisition Lab, Darwin Tree of Life Barcoding collective, Wellcome Sanger Institute Tree of Life Management, Samples and Laboratory team, Wellcome Sanger Institute Scientific Operations: Sequencing Operations, Wellcome Sanger Institute Tree of Life Core Informatics team, Tree of Life Core Informatics collective, Darwin Tree of Life Consortium

**Affiliations:** 1 UK Centre for Ecology & Hydrology, Wallingford, England, UK; 2 Department of Biology, University of Oxford, Oxford, England, UK; 3 Tree of Life, Wellcome Sanger Institute, Hinxton, England, UK

**Keywords:** Photedes minima, Small Dotted Buff, genome sequence, chromosomal, Lepidoptera

## Abstract

We present a genome assembly from a male specimen of *Photedes minima* (Small Dotted Buff; Arthropoda; Insecta; Lepidoptera; Noctuidae). The genome sequence has a total length of 694.66 megabases. Most of the assembly (99.95%) is scaffolded into 31 chromosomal pseudomolecules, including the Z sex chromosome. The mitochondrial genome has also been assembled, with a length of 15.38 kilobases.

## Species taxonomy

Eukaryota; Opisthokonta; Metazoa; Eumetazoa; Bilateria; Protostomia; Ecdysozoa; Panarthropoda; Arthropoda; Mandibulata; Pancrustacea; Hexapoda; Insecta; Dicondylia; Pterygota; Neoptera; Endopterygota; Amphiesmenoptera; Lepidoptera; Glossata; Neolepidoptera; Heteroneura; Ditrysia; Obtectomera; Noctuoidea; Noctuidae; Noctuinae; Apameini; *Photedes*; *Photedes minima* Haworth, 1809 (NCBI:txid988148)

## Background

*Photedes minima*, or the Small Dotted Buff, is a moth from the Noctuidae family with a wingspan of 20–26 mm ( Kimber, 2025). Males typically have a plain buff appearance with faint dark dots and a slight sheen, while females are smaller with markings that are more distinct. Their forewings range from pale ochreous to light brown, often dusted with darker shades, and females usually have a darker median area. The hindwings vary from yellow-orange to greyish near the edges ( Waring *et al.*, 2017).

Distributed across Europe, from the Iberian Peninsula to western Russia ( GBIF Secretariat, 2023), *P. minima* is found in marshes, damp woodlands, and wet meadows ( Kimber, 2025). In the UK, it widespread across Britain and Ireland ( Waring *et al.*, 2017). Its reliance on moist environments ties closely to the availability of its larval host plant, tufted hair-grass ( *Deschampsia cespitosa*) ( Kimber, 2025).

The moth has one generation per year, with adults active from late June to August in a single brood ( Waring *et al.*, 2017). After mating, females lay eggs near host plants and the larvae feed on grass stems before entering diapause over winter. They pupate in an underground cocoon.

**Figure 1.**
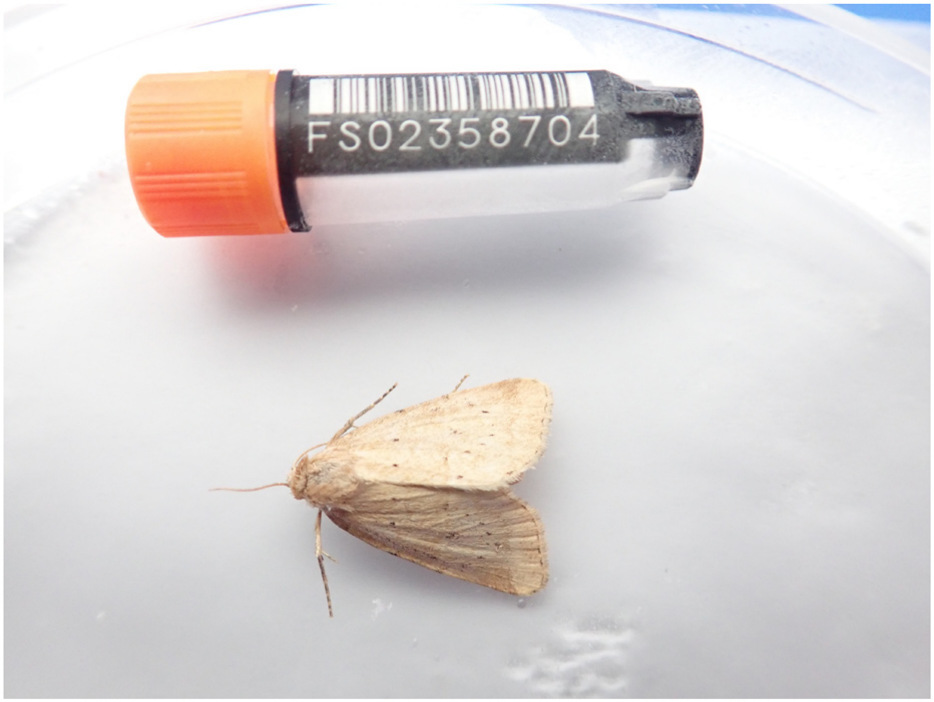
Photograph of the *Photedes minima* (ilPhoMini1) specimen used for genome sequencing.

We present a chromosome-level genome sequence for *Photedes minima*, based on a specimen from Wytham Woods, Oxfordshire, United Kingdom ( Figure 1). The genome was sequenced as part of the Darwin Tree of Life Project and will provide insights into its evolutionary biology and inform conservation strategies ( Blaxter *et al.*, 2022).

## Genome sequence report

### Sequencing data

The genome of a specimen of *Photedes minima* ( Figure 1) was sequenced using Pacific Biosciences single-molecule HiFi long reads, generating 43.48 Gb (gigabases) from 3.17 million reads, which were used to assemble the genome. GenomeScope analysis estimated the haploid genome size at 683.69 Mb, with a heterozygosity of 0.63% and repeat content of 39.57%. These estimates guided expectations for the assembly. Based on the estimated genome size, the sequencing data provided approximately 60 coverage. Hi-C sequencing produced 125.82 Gb from 833.27 million reads, used to scaffold the assembly. RNA sequencing data were also generated and are available in public sequence repositories. Table 1 summarises the specimen and sequencing details.

**Table 1.** Specimen and sequencing data for *Photedes minima*.

Project information			
**Study title**	Photedes minima (small dotted buff)		
**Umbrella BioProject**	PRJEB71302		
**Species**	*Photedes minima*		
**BioSpecimen**	SAMEA7701533		
**NCBI taxonomy ID**	988148		
Specimen information			
**Technology**	**ToLID**	**BioSample accession**	**Organism part**
**PacBio long read sequencing**	ilPhoMini1	SAMEA7701719	whole organism
**Hi-C sequencing**	ilPhoMini2	SAMEA114645394	whole organism
**RNA sequencing**	ilPhoMini2	SAMEA114645394	whole organism
Sequencing information			
**Platform**	**Run accession**	**Read count**	**Base count (Gb)**
**Hi-C Illumina NovaSeq X**	ERR13702755	8.33e+08	125.82
**PacBio Sequel IIe**	ERR12370428	1.36e+06	16.62
**PacBio Sequel IIe**	ERR12370427	1.81e+06	26.86
**RNA Illumina NovaSeq X**	ERR13999093	9.15e+07	13.82

### Assembly statistics

The primary haplotype was assembled, and contigs corresponding to an alternate haplotype were also deposited in INSDC databases. The assembly was improved by manual curation, which corrected 12 misjoins or missing joins and removed 5 haplotypic duplications. These interventions decreased the scaffold count by 4.76%. The final assembly has a total length of 694.66 Mb in 39 scaffolds, with 8 gaps, and a scaffold N50 of 24.27 Mb ( Table 2).

**Table 2.** Genome assembly data for *Photedes minima*.

Genome assembly		
Assembly name	ilPhoMini1.1	
Assembly accession	GCA_964662195.1	
*Alternate haplotype accession*	*GCA_964662255.1*	
Assembly level for primary assembly	chromosome	
Span (Mb)	694.66	
Number of contigs	47	
Number of scaffolds	39	
Longest scaffold (Mb)	33.31	
Assembly metric	Measure	*Benchmark*
Contig N50 length	24.18 Mb	*≥ 1 Mb*
Scaffold N50 length	24.27 Mb	*= chromosome N50*
Consensus quality (QV)	Primary: 68.6; alternate: 66.5; combined: 67.1	*≥ 40*
*k*-mer completeness	Primary: 84.63%; alternate: 84.13%; combined: 98.68%	*≥ 95%*
BUSCO *	C:99.0%[S:98.6%,D:0.5%], F:0.1%,M:0.9%,n:5,286	*S > 90%; D < 5%*
Percentage of assembly assigned to chromosomes	99.95%	*≥ 90%*
Sex chromosomes	Z	*localised homologous pairs*
Organelles	Mitochondrial genome: 15.38 kb	*complete single alleles*

* BUSCO scores based on the lepidoptera_odb10 BUSCO set using version 5.5.0. C = complete [S = single copy, D = duplicated], F = fragmented, M = missing, n = number of orthologues in comparison.

**Figure 2.**
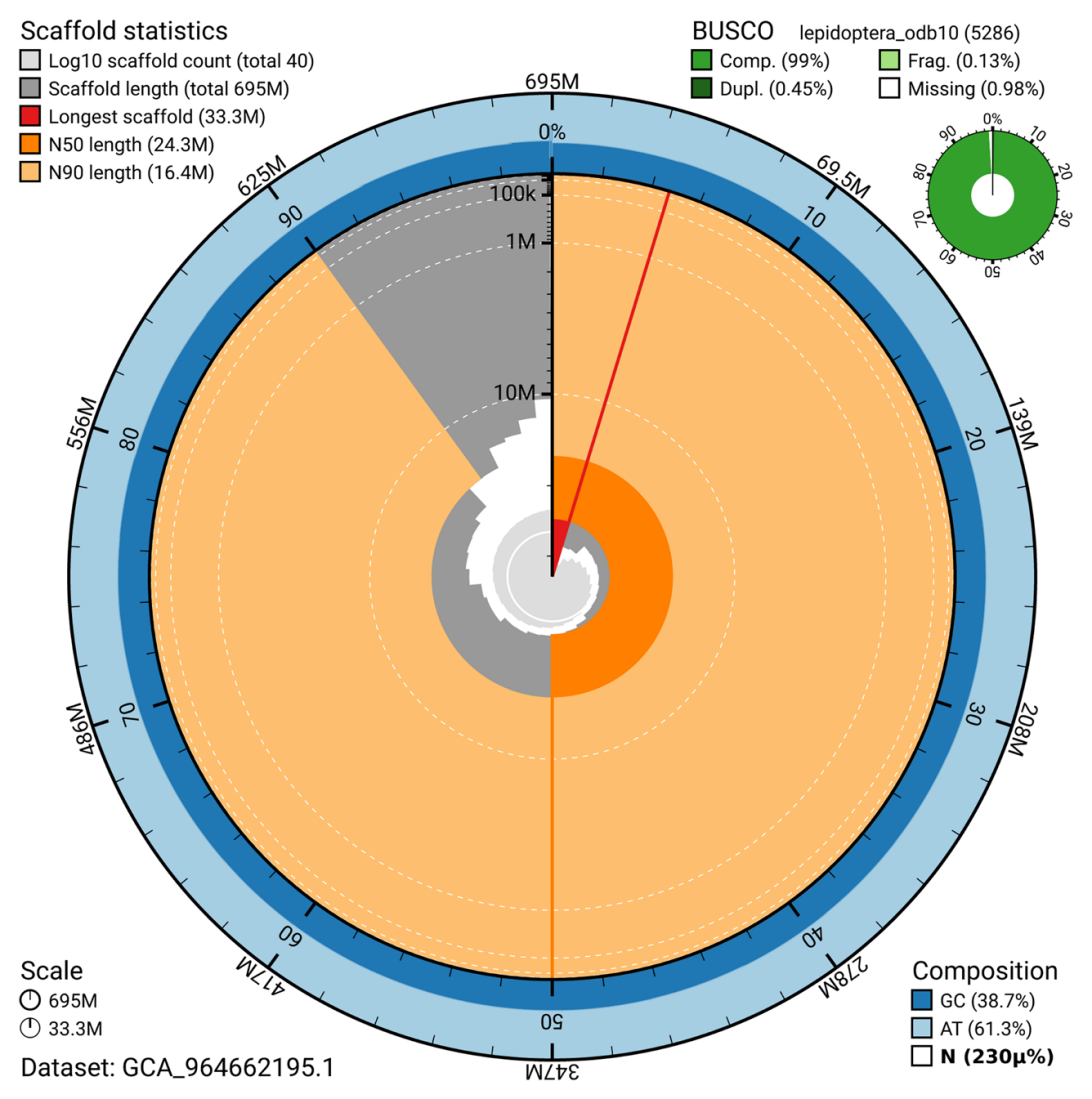
Genome assembly of *Photedes minima*, ilPhoMini1.1: metrics. The BlobToolKit snail plot provides an overview of assembly metrics and BUSCO gene completeness. The circumference represents the length of the whole genome sequence, and the main plot is divided into 1,000 bins around the circumference. The outermost blue tracks display the distribution of GC, AT, and N percentages across the bins. Scaffolds are arranged clockwise from longest to shortest and are depicted in dark grey. The longest scaffold is indicated by the red arc, and the deeper orange and pale orange arcs represent the N50 and N90 lengths. A light grey spiral at the centre shows the cumulative scaffold count on a logarithmic scale. A summary of complete, fragmented, duplicated, and missing BUSCO genes in the lepidoptera_odb10 set is presented at the top right. An interactive version of this figure is available at https://blobtoolkit.genomehubs.org/view/GCA_964662195.1/dataset/GCA_964662195.1/snail.

**Figure 3.**
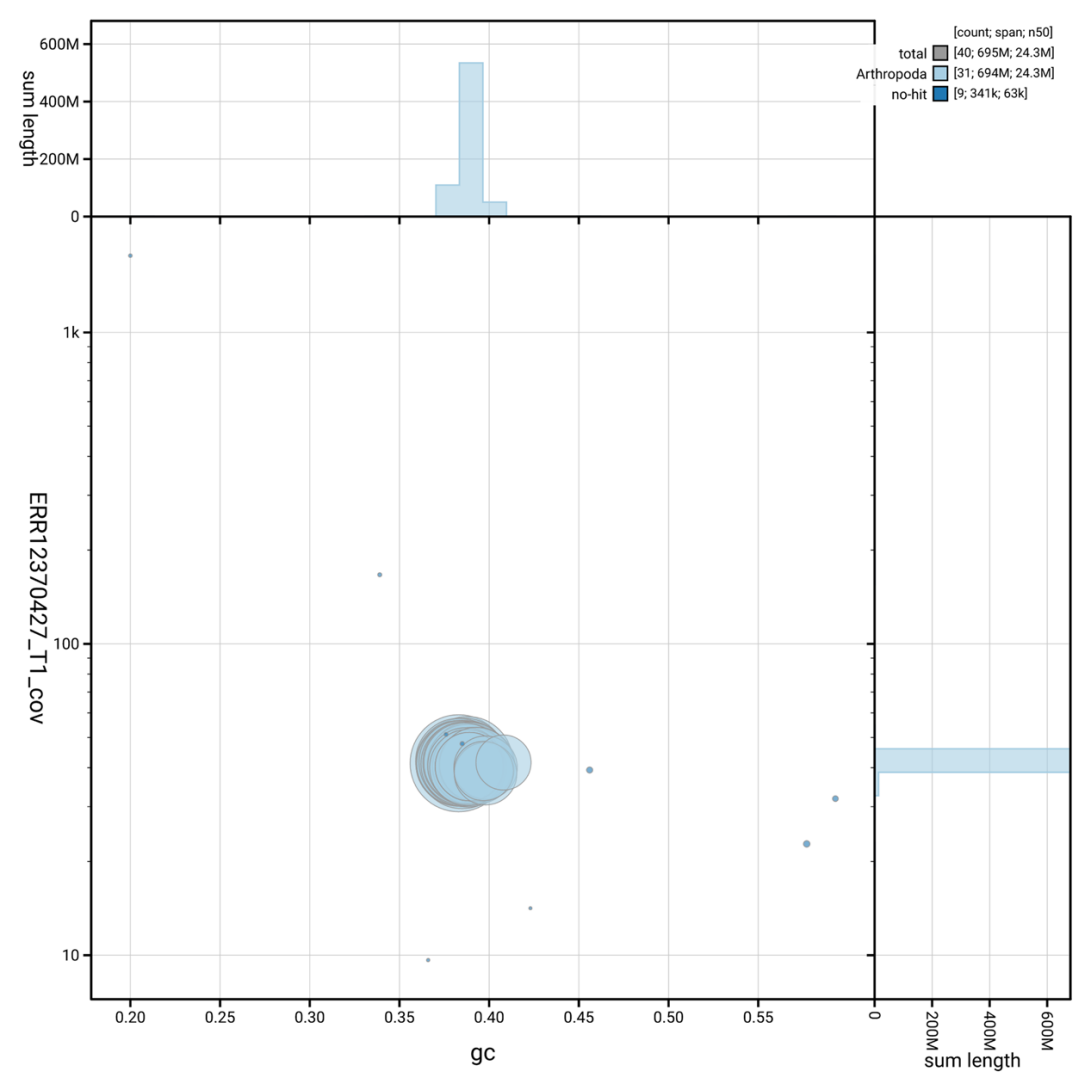
Genome assembly of *Photedes minima*, ilPhoMini1.1: BlobToolKit GC-coverage plot. Blob plot showing sequence coverage (vertical axis) and GC content (horizontal axis). The circles represent scaffolds, with the size proportional to scaffold length and the colour representing phylum membership. The histograms along the axes display the total length of sequences distributed across different levels of coverage and GC content. An interactive version of this figure is available at https://blobtoolkit.genomehubs.org/view/GCA_964662195.1/dataset/GCA_964662195.1/blob.

**Figure 4.**
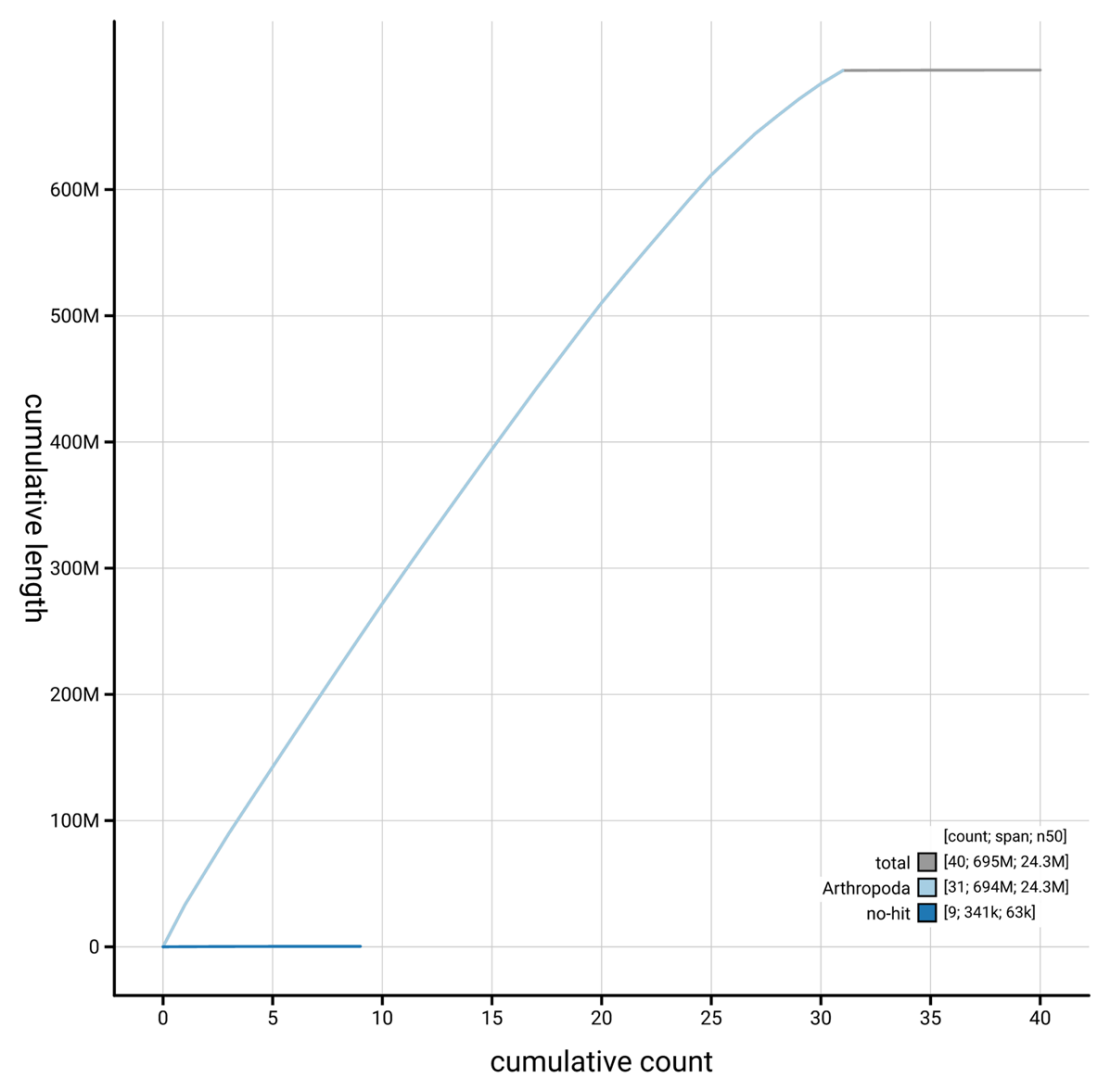
Genome assembly of *Photedes minima,* ilPhoMini1.1: BlobToolKit cumulative sequence plot. The grey line shows cumulative length for all scaffolds. Coloured lines show cumulative lengths of scaffolds assigned to each phylum using the buscogenes taxrule. An interactive version of this figure is available at https://blobtoolkit.genomehubs.org/view/GCA_964662195.1/dataset/GCA_964662195.1/cumulative.

The snail plot in Figure 2 provides a summary of the assembly statistics, indicating the distribution of scaffold lengths and other assembly metrics. Figure 3 shows the distribution of scaffolds by GC proportion and coverage. Figure 4 presents a cumulative assembly plot, with separate curves representing different scaffold subsets assigned to various phyla, illustrating the completeness of the assembly.

**Figure 5.**
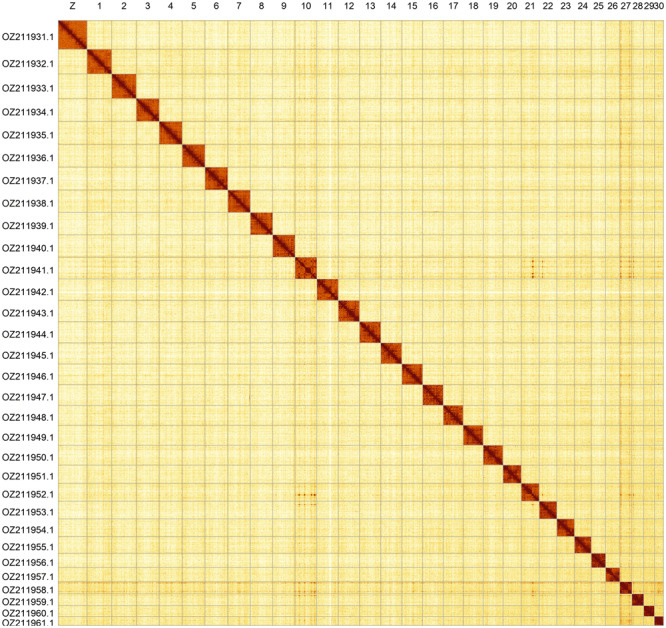
Genome assembly of *Photedes minima*: Hi-C contact map of the ilPhoMini1.1 assembly, generated using PretextSnapshot. Chromosomes are shown in order of size and labelled with chromosome numbers (top) and chromosome accession numbers (left).

Most of the assembly sequence (99.95%) was assigned to 31 chromosomal-level scaffolds, representing 30 autosomes and the Z sex chromosome. These chromosome-level scaffolds, confirmed by Hi-C data, are named according to size ( Figure 5; Table 3). During curation, the Z chromosome was identified based BUSCO gene painting with ancestral Merian elements ( Wright *et al.*, 2024).

**Table 3.** Chromosomal pseudomolecules in the genome assembly of *Photedes minima*, ilPhoMini1.

INSDC accession	Name	Length (Mb)	GC%
OZ211931.1	Z	33.31	38.5
OZ211932.1	1	28.32	39
OZ211933.1	2	28.12	38.5
OZ211934.1	3	26.43	38.5
OZ211935.1	4	26.32	38.5
OZ211936.1	5	26.16	38.5
OZ211937.1	6	26.05	38.5
OZ211938.1	7	25.87	38.5
OZ211939.1	8	25.7	38.5
OZ211940.1	9	25.6	38.5
OZ211941.1	10	25.0	39
OZ211942.1	11	24.63	38.5
OZ211943.1	12	24.3	38.5
OZ211944.1	13	24.27	38.5
OZ211945.1	14	24.18	38.5
OZ211946.1	15	23.82	38.5
OZ211947.1	16	23.76	38.5
OZ211948.1	17	22.94	39
OZ211949.1	18	22.92	39
OZ211950.1	19	22.57	38.5
OZ211951.1	20	21.02	39
OZ211952.1	21	20.52	39.5
OZ211953.1	22	20.27	38.5
OZ211954.1	23	20.17	39
OZ211955.1	24	19.33	39.5
OZ211956.1	25	16.36	39
OZ211957.1	26	16.31	39
OZ211958.1	27	13.93	40
OZ211959.1	28	13.54	40
OZ211960.1	29	12.16	39.5
OZ211961.1	30	10.45	41
OZ211962.1	MT	0.02	20.5

The mitochondrial genome was also assembled. This sequence is included as a contig in the multifasta file of the genome submission and as a standalone record.

### Assembly quality metrics

The estimated Quality Value (QV) and *k*-mer completeness metrics, along with BUSCO completeness scores, were calculated for each haplotype and the combined assembly. The QV reflects the base-level accuracy of the assembly, while *k*-mer completeness indicates the proportion of expected *k*-mers identified in the assembly. BUSCO scores provide a measure of completeness based on benchmarking universal single-copy orthologues.

The combined primary and alternate assemblies achieve an estimated QV of 67.1. The *k*-mer completeness is 84.63% for the primary haplotype and 84.13% for the alternate haplotype; and 98.68% for the combined primary and alternate assemblies. BUSCO v.5.5.0 analysis using the lepidoptera_odb10 reference set ( *n* = 5,286) identified 99.0% of the expected gene set (single = 98.6%, duplicated = 0.5%).

Table 2 provides assembly metric benchmarks adapted from Rhie *et al.* (2021) and the Earth BioGenome Project Report on Assembly Standards September 2024. The primary assembly achieves the EBP reference standard of **7.C.Q68**.

## Methods

### Sample acquisition and DNA barcoding

The specimen used for genome sequencing was an adult male *Photedes minima* (specimen ID Ox000672, ToLID ilPhoMini1), collected from Wytham Woods, Oxfordshire, United Kingdom (latitude 51.772, longitude –1.338) on 2020-07-20, using a light trap. The specimen was collected and identified by Douglas Boyes (University of Oxford) and preserved on dry ice. A second specimen was used for Hi-C and RNA sequencing (specimen ID Ox003713, ToLID ilPhoMini2), was collected from the same location on 2023-06-20. The specimen was collected and identified by Liam Crowley (University of Oxford) and preserved on dry ice.

The initial identification was verified by an additional DNA barcoding process according to the framework developed by Twyford *et al.* (2024). A small sample was dissected from each specimen and stored in ethanol, while the remaining parts were shipped on dry ice to the Wellcome Sanger Institute (WSI) ( Pereira *et al.*, 2022). The tissue was lysed, the COI marker region was amplified by PCR, and amplicons were sequenced and compared to the BOLD database, confirming the species identification ( Crowley *et al.*, 2023). Following whole genome sequence generation, the relevant DNA barcode region was also used alongside the initial barcoding data for sample tracking at the WSI ( Twyford *et al.*, 2024). The standard operating procedures for Darwin Tree of Life barcoding have been deposited on protocols.io ( Beasley *et al.*, 2023).

Metadata collection for samples adhered to the Darwin Tree of Life project standards described by Lawniczak *et al.* (2022).

### Nucleic acid extraction

The workflow for high molecular weight (HMW) DNA extraction at the Wellcome Sanger Institute (WSI) Tree of Life Core Laboratory includes a sequence of procedures: sample preparation and homogenisation, DNA extraction, fragmentation and purification ( Howard *et al.*, 2025). Detailed protocols are available on protocols.io ( Denton *et al.*, 2023b). The ilPhoMini1 sample was prepared for DNA extraction by weighing and dissecting it on dry ice ( Jay *et al.*, 2023). Tissue from the whole organism was homogenised using a PowerMasher II tissue disruptor ( Denton *et al.*, 2023a). HMW DNA was extracted using the Automated MagAttract v1 protocol ( Sheerin *et al.*, 2023). DNA was sheared into an average fragment size of 12–20 kb in a Megaruptor 3 system ( Todorovic *et al.*, 2023). Sheared DNA was purified by solid-phase reversible immobilisation, using AMPure PB beads to eliminate shorter fragments and concentrate the DNA ( Strickland *et al.*, 2023). The concentration of the sheared and purified DNA was assessed using a Nanodrop spectrophotometer and a Qubit Fluorometer using the Qubit dsDNA High Sensitivity Assay kit. The fragment size distribution was evaluated by running the sample on the FemtoPulse system.

RNA was extracted from whole organism tissue of ilPhoMini2 in the Tree of Life Laboratory at the WSI using the RNA Extraction: Automated MagMax™ *mir*Vana protocol ( do Amaral *et al.*, 2023). The RNA concentration was assessed using a Nanodrop spectrophotometer and a Qubit Fluorometer using the Qubit RNA Broad-Range Assay kit. Analysis of the integrity of the RNA was done using the Agilent RNA 6000 Pico Kit and Eukaryotic Total RNA assay.

### Hi-C sample preparation and crosslinking

Hi-C data were generated from the whole organism of the ilPhoMini2 sample using the Arima-HiC v2 kit (Arima Genomics) with 20–50 mg of frozen tissue (stored at –80 °C). As per manufacturer’s instructions, tissue was fixed, and the DNA crosslinked using a TC buffer with 22% formaldehyde concentration, and a final formaldehyde concentration of 2%. The tissue was then homogenised using the Diagnocine Power Masher-II. The crosslinked DNA was digested using a restriction enzyme master mix, then biotinylated and ligated. A clean up was performed with SPRIselect beads prior to library preparation. DNA concentration was quantified using the Qubit Fluorometer v4.0 (Thermo Fisher Scientific) and Qubit HS Assay Kit, and sample biotinylation percentage was estimated using the Arima-HiC v2 QC beads.

### Library preparation and sequencing

Library preparation and sequencing were performed at the WSI Scientific Operations core.

***PacBio HiFi***

At a minimum, samples were required to have an average fragment size exceeding 8 kb and a total mass over 400 ng to proceed to the low-input SMRTbell Prep Kit 3.0 protocol (Pacific Biosciences), depending on genome size and sequencing depth required. Libraries were prepared using the SMRTbell Prep Kit 3.0 as per the manufacturer's instructions. The kit includes the reagents required for end repair/A-tailing, adapter ligation, post-ligation SMRTbell bead cleanup, and nuclease treatment. Size-selection and clean-up were carried out using diluted AMPure PB beads (Pacific Biosciences). DNA concentration was quantified using the Qubit Fluorometer v4.0 (ThermoFisher Scientific) with Qubit 1X dsDNA HS assay kit and the final library fragment size analysis was carried out using the Agilent Femto Pulse Automated Pulsed Field CE Instrument (Agilent Technologies) and the gDNA 55kb BAC analysis kit.

Samples were sequenced using the Sequel IIe system (Pacific Biosciences, California, USA). The concentration of the library loaded onto the Sequel IIe was in the range 40–135 pM. The SMRT link software, a PacBio web-based end-to-end workflow manager, was used to set-up and monitor the run, as well as perform primary and secondary analysis of the data upon completion.

***Hi-C***

For Hi-C library preparation, the biotinylated DNA constructs were fragmented using a Covaris E220 sonicator and size-selected to 400–600 bp using SPRISelect beads. DNA was then enriched using Arima-HiC v2 Enrichment beads. The NEBNext Ultra II DNA Library Prep Kit (New England Biolabs) was used for end repair, A-tailing, and adapter ligation, following a modified protocol in which library preparation is carried out while the DNA remains bound to the enrichment beads. PCR amplification was performed using KAPA HiFi HotStart mix and custom dual-indexed adapters (Integrated DNA Technologies) in a 96-well plate format. Depending on sample concentration and biotinylation percentage determined at the crosslinking stage, samples were amplified for 10–16 PCR cycles. Post-PCR clean-up was carried out using SPRISelect beads. The libraries were quantified using the Accuclear Ultra High Sensitivity dsDNA Standards Assay kit (Biotium) and normalised to 10 ng/μL before sequencing. Hi-C sequencing was performed on the Illumina NovaSeq X instrument using 150 bp paired-end reads.

***RNA***

Poly(A) RNA-Seq libraries were constructed using the NEB Ultra II RNA Library Prep kit, following the manufacturer’s instructions. RNA sequencing was performed on the Illumina NovaSeq X instrument.

### Genome assembly, curation and evaluation

***Assembly***

Prior to assembly of the PacBio HiFi reads, a database of *k*-mer counts ( *k* = 31) was generated from the filtered reads using FastK. GenomeScope2 ( Ranallo-Benavidez *et al.*, 2020) was used to analyse the *k*-mer frequency distributions, providing estimates of genome size, heterozygosity, and repeat content.

The HiFi reads were first assembled using Hifiasm ( Cheng *et al.*, 2021) with the --primary option. Haplotypic duplications were identified and removed using purge_dups ( Guan *et al.*, 2020). The Hi-C reads ( Rao *et al.*, 2014) were mapped to the primary contigs using bwa-mem2 ( Vasimuddin *et al.*, 2019), and the contigs were scaffolded in YaHS ( Zhou *et al.*, 2023) using the --break option for handling potential misassemblies. The scaffolded assemblies were evaluated using Gfastats ( Formenti *et al.*, 2022), BUSCO ( Manni *et al.*, 2021) and MERQURY.FK ( Rhie *et al.*, 2020).

The mitochondrial genome was assembled using MitoHiFi ( Uliano-Silva *et al.*, 2023), which runs MitoFinder ( Allio *et al.*, 2020) and uses these annotations to select the final mitochondrial contig and to ensure the general quality of the sequence.

***Assembly curation***

The assembly was decontaminated using the Assembly Screen for Cobionts and Contaminants (ASCC) pipeline. Flat files and maps used in curation were generated via the TreeVal pipeline ( Pointon *et al.*, 2023). Manual curation was conducted primarily in PretextView ( Harry, 2022) and HiGlass ( Kerpedjiev *et al.*, 2018), with additional insights provided by JBrowse2 ( Diesh *et al.*, 2023). Scaffolds were visually inspected and corrected as described by Howe *et al.* (2021). Any identified contamination, missed joins, and mis-joins were amended, and duplicate sequences were tagged and removed. Sex chromosomes were identified by Merian element painting. The curation process is documented at https://gitlab.com/wtsi-grit/rapid-curation.

***Assembly quality assessment***

The Merqury.FK tool ( Rhie *et al.*, 2020), run in a Singularity container ( Kurtzer *et al.*, 2017), was used to evaluate *k*-mer completeness and assembly quality for the primary and alternate haplotypes using the *k*-mer databases ( *k* = 31) computed prior to genome assembly. The analysis outputs included assembly QV scores and completeness statistics.

The genome was analysed in the blobtoolkit pipeline, a Nextflow ( Di Tommaso *et al.*, 2017) port of the previous Snakemake Blobtoolkit pipeline ( Challis *et al.*, 2020). It aligns the PacBio reads in SAMtools ( Danecek *et al.*, 2021) and minimap2 ( Li, 2018) and generates coverage tracks for regions of fixed size. In parallel, it queries the GoaT database ( Challis *et al.*, 2023) to identify all matching BUSCO lineages to run BUSCO ( Manni *et al.*, 2021). For the three domain-level BUSCO lineages, the pipeline aligns the BUSCO genes to the UniProt Reference Proteomes database ( Bateman *et al.*, 2023) with DIAMOND blastp ( Buchfink *et al.*, 2021). The genome is also divided into chunks according to the density of the BUSCO genes from the closest taxonomic lineage, and each chunk is aligned to the UniProt Reference Proteomes database using DIAMOND blastx. Genome sequences without a hit are chunked using seqtk and aligned to the NT database with blastn ( Altschul *et al.*, 1990). The blobtools suite combines all these outputs into a blobdir for visualisation.

The blobtoolkit pipeline was developed using nf-core tooling ( Ewels *et al.*, 2020) and MultiQC ( Ewels *et al.*, 2016), relying on the Conda package manager, the Bioconda initiative ( Grüning *et al.*, 2018), the Biocontainers infrastructure ( da Veiga Leprevost *et al.*, 2017), as well as the Docker ( Merkel, 2014) and Singularity ( Kurtzer *et al.*, 2017) containerisation solutions.

Table 4 contains a list of relevant software tool versions and sources.

**Table 4.** Software tools: versions and sources.

Software tool	Version	Source
BLAST	2.14.0	ftp://ftp.ncbi.nlm.nih.gov/blast/executables/blast+/
BlobToolKit	4.3.9	https://github.com/blobtoolkit/blobtoolkit
BUSCO	5.5.0	https://gitlab.com/ezlab/busco
bwa-mem2	2.2.1	https://github.com/bwa-mem2/bwa-mem2
DIAMOND	2.1.8	https://github.com/bbuchfink/diamond
fasta_windows	0.2.4	https://github.com/tolkit/fasta_windows
FastK	666652151335353eef2fcd58880bcef5bc2928e1	https://github.com/thegenemyers/FASTK
Gfastats	1.3.6	https://github.com/vgl-hub/gfastats
GoaT CLI	0.2.5	https://github.com/genomehubs/goat-cli
Hifiasm	0.19.8-r603	https://github.com/chhylp123/hifiasm
HiGlass	44086069ee7d4d3f6f3f0012569789ec138f42b84aa44357826c0b6753eb28de	https://github.com/higlass/higlass
MerquryFK	d00d98157618f4e8d1a9190026b19b471055b22e	https://github.com/thegenemyers/MERQURY.FK
Minimap2	2.24-r1122	https://github.com/lh3/minimap2
MitoHiFi	3	https://github.com/marcelauliano/MitoHiFi
MultiQC	1.14, 1.17, and 1.18	https://github.com/MultiQC/MultiQC
Nextflow	23.10.0	https://github.com/nextflow-io/nextflow
PretextView	0.2.5	https://github.com/sanger-tol/PretextView
PretextSnapshot	-	https://github.com/sanger-tol/PretextSnapshot
samtools	1.19.2	https://github.com/samtools/samtools
sanger-tol/ascc	0.1.0	https://github.com/sanger-tol/ascc
sanger-tol/blobtoolkit	0.6.0	https://github.com/sanger-tol/blobtoolkit
Seqtk	1.3	https://github.com/lh3/seqtk
Singularity	3.9.0	https://github.com/sylabs/singularity
TreeVal	1.2.0	https://github.com/sanger-tol/treeval
YaHS	1.2a.2	https://github.com/c-zhou/yahs
GenomeScope2.0	2.0.1	https://github.com/tbenavi1/genomescope2.0

### Wellcome Sanger Institute – Legal and Governance

The materials that have contributed to this genome note have been supplied by a Darwin Tree of Life Partner. The submission of materials by a Darwin Tree of Life Partner is subject to the **‘Darwin Tree of Life Project Sampling Code of Practice’**, which can be found in full on the Darwin Tree of Life website here. By agreeing with and signing up to the Sampling Code of Practice, the Darwin Tree of Life Partner agrees they will meet the legal and ethical requirements and standards set out within this document in respect of all samples acquired for, and supplied to, the Darwin Tree of Life Project.

Further, the Wellcome Sanger Institute employs a process whereby due diligence is carried out proportionate to the nature of the materials themselves, and the circumstances under which they have been/are to be collected and provided for use. The purpose of this is to address and mitigate any potential legal and/or ethical implications of receipt and use of the materials as part of the research project, and to ensure that in doing so we align with best practice wherever possible. The overarching areas of consideration are:

•   Ethical review of provenance and sourcing of the material

•   Legality of collection, transfer and use (national and international)

Each transfer of samples is further undertaken according to a Research Collaboration Agreement or Material Transfer Agreement entered into by the Darwin Tree of Life Partner, Genome Research Limited (operating as the Wellcome Sanger Institute), and in some circumstances other Darwin Tree of Life collaborators.

## Data Availability

European Nucleotide Archive: Photedes minima (small dotted buff). Accession number PRJEB71302; https://identifiers.org/ena.embl/PRJEB71302. The genome sequence is released openly for reuse. The *Photedes minima* genome sequencing initiative is part of the Darwin Tree of Life Project (PRJEB40665), the Sanger Institute Tree of Life Programme (PRJEB43745) and Project Psyche (PRJEB71705). All raw sequence data and the assembly have been deposited in INSDC databases. The genome will be annotated using available RNA-Seq data and presented through the Ensembl pipeline at the European Bioinformatics Institute. Raw data and assembly accession identifiers are reported in Table 1 and Table 2.

## Author information

Members of the University of Oxford and Wytham Woods Genome Acquisition Lab are listed here: https://doi.org/10.5281/zenodo.12157525.

Members of the Darwin Tree of Life Barcoding collective are listed here: https://doi.org/10.5281/zenodo.12158331.

Members of the Wellcome Sanger Institute Tree of Life Management, Samples and Laboratory team are listed here: https://doi.org/10.5281/zenodo.12162482.

Members of Wellcome Sanger Institute Scientific Operations: Sequencing Operations are listed here: https://doi.org/10.5281/zenodo.12165051.

Members of the Wellcome Sanger Institute Tree of Life Core Informatics team are listed here: https://doi.org/10.5281/zenodo.12160324.

Members of the Tree of Life Core Informatics collective are listed here: https://doi.org/10.5281/zenodo.12205391.

Members of the Darwin Tree of Life Consortium are listed here: https://doi.org/10.5281/zenodo.4783558.
