# Supplementary material for: The genome sequence of the Small Dotted Buff moth, Photedes minima Haworth, 1809
Source: Wellcome Open Res. 2025 Jun 2;10:299. [Version 1] doi: 10.12688/wellcomeopenres.24264.1 (PMC12284473; doi:10.12688/wellcomeopenres.24264.1)
Supplement: Peer review history

# Reviewer report: Reviewer response for version 1

**DOI:** 10.21956/wellcomeopenres.26761.r125566

**Published:** 2025-07-22

**Authors:** Sivasankaran Kuppusamy

**Affiliations:** 1 Loyola College, Chennai, India

Authors have done the chromosome-level genome assembly of Small Dotted Buff moth, *Photedes minima *Haworth, 1809. They generated 183.12 Gb sequence data through different sequencing platforms. Then assembled the sequences and obtained a total length of 696.66 megabases. Authors have identified 31 chromosomal pseudomolecules. The comprehensive data is useful for the phylogenomic studies of moths.

**Minor comments**

 The first sentence of the Background may include the describer’s name for the species i.e., *Photedes minima *Haworth, 1809

 The second paragraph of the background first sentence may be written as “ *P. minima *is distributed across Europe, from the Iberian Peninsula to western Russia (GBIF Secretariate, 2023) and is usually found in marshes, damp woodlands and wet meadows (Kimber, 2025)”. 

 The first sentence of the second paragraph can be modified as “The moth has an univoltine, with adults active from late June to August………………..

 We present a chromosome-level genome sequence for *P. minima*………………

 Throughout the text, the author consistently used the genus' full name, *Photedes minima*. Start with the full genus name once, if possible, then shorten it to *P. minima*.

 Authors have produced 183.12 Gb comprehensive sequences through the Hi-C illumina and PacBio platforms. After the assembly they received 694.66 megabases. Through the genome annotation the authors haven’t listed the protein-coding genes, non-coding genes and gene transcripts.

 The research article was well prepared, and the manuscript meets the necessary scientific standard and is suitable for indexing.

Are sufficient details of methods and materials provided to allow replication by others?

Yes

Is the rationale for creating the dataset(s) clearly described?

Yes

Are the datasets clearly presented in a useable and accessible format?

Yes

Are the protocols appropriate and is the work technically sound?

Yes

Reviewer Expertise:

Phylogenetic analysis of Noctuoidea moths using mitogenome sequence

I confirm that I have read this submission and believe that I have an appropriate level of expertise to confirm that it is of an acceptable scientific standard.

# Reviewer report: Reviewer response for version 1

**DOI:** 10.21956/wellcomeopenres.26761.r125571

**Published:** 2025-07-07

**Authors:** Željko Tomanović

**Affiliations:** 1 Serbia University of Belgrade, Belgrade, Serbia

Boyes et al. presented a genome assembly of *Photedes minima* (Small Dotted Buff; Arthropoda; Insecta; Lepidoptera; Noctuidae), a species with a wide European distribution. The authors briefly presented the biology and ecology of the species. The genome sequence has a total length of 694.66 megabases, which are distributed over 31 chromosomes, including 30 autosomes and the sex chromosome Z. The mitochondrial genome was also assembled. The authors have presented the assembly statistics and all the methods used in detail. Ms is very well written.

Are sufficient details of methods and materials provided to allow replication by others?

Yes

Is the rationale for creating the dataset(s) clearly described?

Yes

Are the datasets clearly presented in a useable and accessible format?

Yes

Are the protocols appropriate and is the work technically sound?

Yes

Reviewer Expertise:

biodiversity, phylogenetics, ecology

I confirm that I have read this submission and believe that I have an appropriate level of expertise to confirm that it is of an acceptable scientific standard.
